# Examining the use of the FIGO Nutrition Checklist in routine antenatal practice: multistakeholder feedback to implementation

**DOI:** 10.1002/ijgo.13323

**Published:** 2020-09-07

**Authors:** Sarah Louise Killeen, Shauna L. Callaghan, Chandni Maria Jacob, Mark A. Hanson, Fionnuala M. McAuliffe

**Affiliations:** ^1^ UCD Perinatal Research Centre School of Medicine University College Dublin National Maternity Hospital Dublin Ireland; ^2^ School of Nursing, Midwifery and Health Systems University College Dublin Dublin Ireland; ^3^ Institute of Developmental Sciences University of Southampton Southampton UK; ^4^ NIHR Southampton Biomedical Research Centre University Hospital Southampton Southampton UK

**Keywords:** Acceptability study, Antenatal care, Feasibility study, FIGO Nutrition Checklist, Gestational weight gain, Nutrition, Obesity, Pregnancy, Screening tool

## Abstract

**Objective:**

To gain insights from pregnant women and obstetricians on the utility of the FIGO Nutrition Checklist in antenatal practice.

**Methods:**

Women were recruited from the antenatal department of a large tertiary‐level university maternity hospital in Dublin, Ireland, between October and December 2019. Participants completed the FIGO Nutrition Checklist before their routine antenatal appointment. Obstetricians and women were encouraged to discuss the FIGO Nutrition Checklist during the clinical visit. Completed FIGO Nutrition Checklists were collected after appointments. Acceptability was assessed through questionnaires.

**Results:**

The majority (80.0%) of women answered “No” to at least one diet quality question, indicating a potential nutritional risk. While none of the participating obstetricians routinely discussed nutrition with women, all agreed that using the Checklist encouraged them to address nutrition with pregnant women. Nearly every woman (99.0%) found the Checklist quick to complete; however, all participating obstetricians felt there was not enough time to discuss it in routine practice. Despite this, most obstetricians and pregnant women recommended the FIGO Nutrition Checklist for use.

**Conclusion:**

The FIGO Nutrition Checklist is acceptable for use in routine antenatal practice in tertiary care settings. It helped identify potentially at‐risk women during early pregnancy and facilitated conversations related to optimum diet.

## Abstract 2

The FIGO Nutrition Checklist is acceptable to pregnant women and may capture high‐risk weight or dietary practices in pregnancy that would otherwise go untreated.

## 1 Introduction

Addressing nutrition in women of reproductive age offers a unique opportunity to influence global health targets for noncommunicable disease (NCD) reduction and management.^1^, ^2^ Dietary interventions in pregnancy are also likely to be cost‐effective.^3^ Despite this, diet is not universally addressed by obstetricians and gynecologists as a routine part of maternity care.^4^, ^5^ Many women do not meet dietary recommendations during pregnancy, despite identifying healthy eating as a personal priority.^6^ Pregnancy has been argued to provide a “teachable moment,” when women may be more motivated to undertake diet and lifestyle changes.^7^, ^8^ Research suggests that healthcare professionals are the most important source of nutrition information in pregnancy and that brief interventions delivered by healthcare professionals as part of routine care can promote positive health behaviours.^9^

The American College of Obstetricians and Gynecologists (ACOG) recommends the development and use of clinical checklists in obstetrics and gynecology.^10^ In 2015, FIGO (the International Federation of Gynecology and Obstetrics) developed a simple nutritional questionnaire called the FIGO Nutrition Checklist. The purpose of the Checklist is to collect basic information from women about nutrition and weight through a series of short questions. These questions are intended to facilitate healthy conversations between healthcare professionals and women before and during pregnancy. Aspects of dietary intake addressed in the FIGO Nutrition Checklist include reported frequency of consumption of different food groups. The FIGO Nutrition Checklist is given as supporting information S1.

The limited available research suggests that simple self‐assessment tools based on food groups are sufficiently accurate for clinical practice and have acceptable agreement with more robust measures of dietary assessment such as seven‐day weighed records.^11^ Acceptability is an important consideration for any new clinical intervention.^12^ The aim of the present study was therefore to investigate the acceptability and feasibility of using the FIGO Nutrition Checklist in routine antenatal practice.

## 2 Materials and methods

This study was a prospective pilot study with a convenient sample of women attending for routine antenatal care. Details of the methodology are reported following the CONSORT extension for pilot and feasibility trials.^13^ The study took place during routine antenatal clinics in the outpatient department of a tertiary‐level university maternity hospital, the National Maternity Hospital, in Dublin between October 7, 2019 and December 12, 2019. Full ethical approval was obtained from the hospital ethics committee (EC202019). All women and healthcare professionals were provided with an information sheet and were asked to read this prior to providing consent to take part. Written informed consent was obtained for study participation.

All English‐speaking women of any gestation or parity attending the chosen pilot clinics were eligible to take part. All routine antenatal clinics were eligible for inclusion in the pilot study; however, specialist clinics including diabetes clinics were excluded as lifestyle advice is incorporated as part of routine management for these pregnancies. Pilot clinics were chosen based on agreement with individual consultants, staff availability, and the number of women booked into the clinic. During the pilot clinics, a member of the research team (SLK/SC) was on‐site to answer any questions or concerns from the women or healthcare professionals. Within 3 months, the aim was to complete at least one clinic a week. However, actual clinic numbers varied based on these factors.

Women were asked to complete the FIGO Nutrition Checklist autonomously. The Checklist includes questions on diet, self‐reported height and weight, and markers of micronutrient status. Diet quality is assessed through six “Yes” or “No” questions and women are asked to outline if they follow a special diet.

Acceptability of the FIGO Nutrition Checklist was assessed using two distinct, specifically designed, self‐administered questionnaires for pregnant women and healthcare professionals, respectively. The aspects of acceptability assessed in this study were informed by the theoretical framework of Sekhon et al.^12^ These included affective attitude, burden, self‐efficacy, perceived effectiveness, and intervention coherence. Expert validation of the questionnaires was conducted with a multidisciplinary group including dietitians, midwives, obstetricians, and public health practitioners. For pregnant women, the acceptability of completing the FIGO Nutrition Checklist autonomously was assessed using six questions, graded with a Likert scale. Women were also asked whether they discussed the FIGO Nutrition Checklist with their doctor and to provide feedback on this if applicable, again with specifically designed questions using a Likert scale. The questionnaire for healthcare professionals also assessed acceptability using a Likert scale; however, these questions were focused on how the FIGO Nutrition Checklist affected their practice. Additional data collected included medical title, number of years’ clinical experience, and whether they used the FIGO Nutrition Checklist during the clinic.

Women were given the FIGO Nutrition Checklist and feedback questionnaire in the waiting room of the outpatient department, before meeting their healthcare professional. They were encouraged to complete the Checklist while waiting for their appointment and to discuss any diet or nutritional issues highlighted by the Checklist with their healthcare professional. Women attending clinics were requested to complete their feedback questionnaire after their appointment with their healthcare professional. Completed Checklists and questionnaires were collected after the same antenatal visit. The feedback from healthcare professionals was collected at the end of their clinic.

A power calculation was not performed for this study as it was a pilot study. A convenient sample of women in the waiting room of the antenatal clinics was obtained. The women in the present study were similar in age and body mass index (BMI, calculated as weight in kilograms divided by the square of height in meters) to participants who took part in previous larger randomized controlled trials completed by our research group.^14^ Women are included from all trimesters of pregnancy (11–40 weeks) and the present sample is therefore representative of the women usually attending these antenatal services.

Statistical analysis was carried out using SPSS version 20 (IBM, Armonk, NY, USA). Descriptive statistics were generated including mean, median, and frequencies, as appropriate. All variables were assessed for distribution of normality through a visual analysis of histograms and normality tests. Independent *t* tests were used to compare means of variables that are normally distributed, while Mann‐Whitney *U* tests were performed to assess differences in nonparametric data. The level of significance was set at *P*<0.05.

## 3 Results

A total of 125 women took part in the pilot study over the course of eight clinics and, of these, we had complete data on the nutrition‐related questions for 105 women (Table 1).

**Table 1:** Age and gestation of pregnant women included in the study (n=105).^a^

Characteristics	Total (n=105)	Answered “Yes” to all diet quality questions (n=21)	Answered “No” to at least one diet quality question (n=84)	*P* value^b^
Age, y	33.3 ± 4.2	31.7 ± 5.0	33.7 ± 4.0	0.05
Gestational age, wk	27.4 (21.0–36.0)	27.0 (20.0–35.5)	29.0 (20.5–36.0)	0.74

^a^ Values are given as mean ± SD and median (interquartile range) unless otherwise indicated.

^b^ *P* values determined using Mann‐Whitney *U* and independent sample *t* tests.

### 3.1 Dietary practices

Of 105 women, 14 (13.3%) reported following a special diet. This was mostly a vegetarian diet (n=7, 6.7%) followed by diets with certain restricted foods (e.g. to control allergy or intolerance to dairy, nuts, or other foods, or for personal preferences) reported by 3 (2.9%) women and diets for the management of conditions such as diabetes, hemochromatosis, or irritable bowel syndrome (n=4, 3.8%).

**Figure 1:**
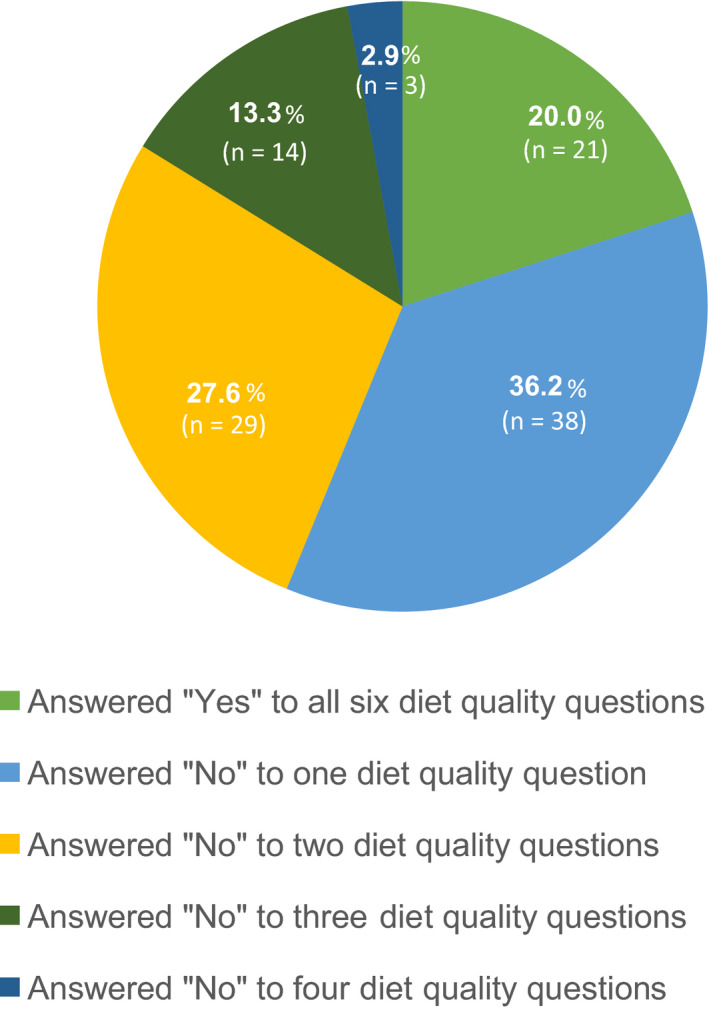
Answers of participating pregnant women (n=105) to the six diet quality questions in the FIGO Nutrition Checklist.

**Figure 2:**
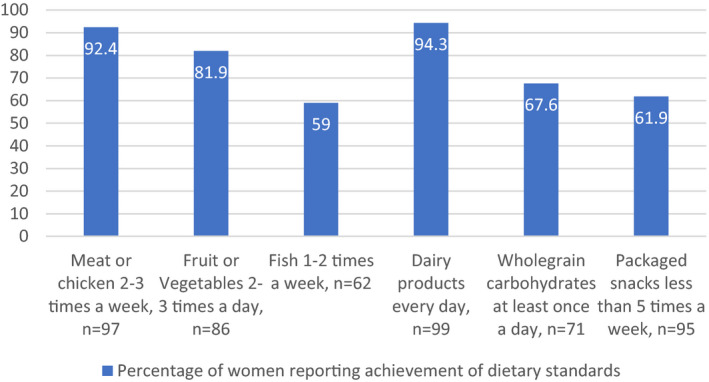
Self‐reported dietary intakes of food groups in pregnant women (n=105).

Figure 1 shows the percentage of women who reported potentially at‐risk dietary practices, as defined by answering “No” to one or more of the diet quality questions outlined in the FIGO Nutrition Checklist. Details of the percentage of women who answered “Yes” to each of the six questions separately can be seen in Figure 2. Importantly, over a quarter of women (n=75) did not know their own weight and height and therefore did not complete this section of the FIGO Nutrition Checklist. Of those, the median BMI was 25.7 (23.0–30.7).

**Figure 3:**
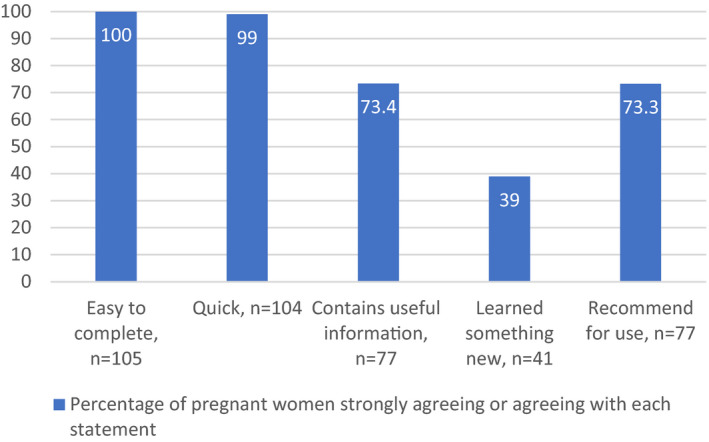
Feedback from pregnant women (n=105) on autonomously completing the FIGO Nutrition Checklist.

Results for the feedback questionnaire are shown in Figure 3. In addition, 97 (92.4%) pregnant women agreed or strongly agreed that they had thought about diet for pregnancy before seeing the FIGO Nutrition Checklist.

**Figure 4:**
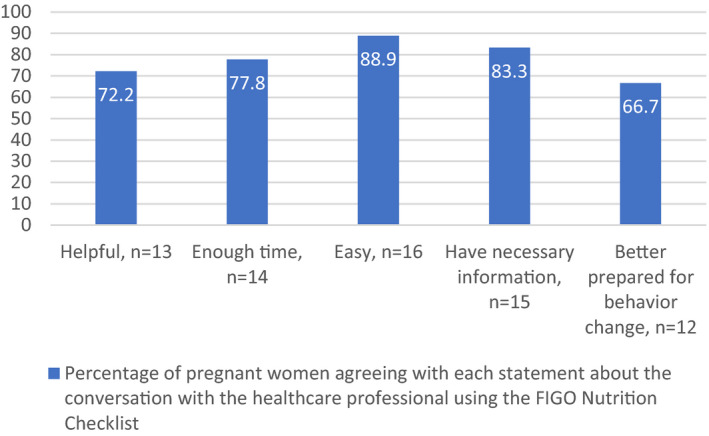
Feedback from pregnant women on discussing the FIGO Nutrition Checklist in clinic (n=18).

Finally, 20 (19.04%) women reported discussing the FIGO Nutrition Checklist with their obstetrician or midwife during their routine antenatal visit and complete feedback data were collected from 18 women. Figure 4 shows the feedback from these women on the experience of discussing the FIGO Nutrition Checklist with their healthcare professional as part of their standard antenatal clinic appointment.

### 3.2 Feedback from healthcare professionals

We surveyed three obstetricians who each piloted the use of the FIGO Nutrition Checklist over the study period. They all had at least two years’ clinical experience and saw approximately 25 women per clinic. None of the obstetricians surveyed said that they currently routinely discuss nutrition with their patients as standard. All obstetricians agreed that nutrition discussions are important in pregnancy. Two obstetricians reported that conversations on nutrition and weight are difficult to initiate in routine practice. Furthermore, only one obstetrician felt confident about discussing nutrition. All three obstetricians agreed that using the FIGO Nutrition Checklist meant they discussed weight and nutrition with more women that they would normally. Two of the three obstetricians agreed that the FIGO Nutrition Checklist would be a beneficial tool for clinical practice and recommend it is used.

**Figure 5:**
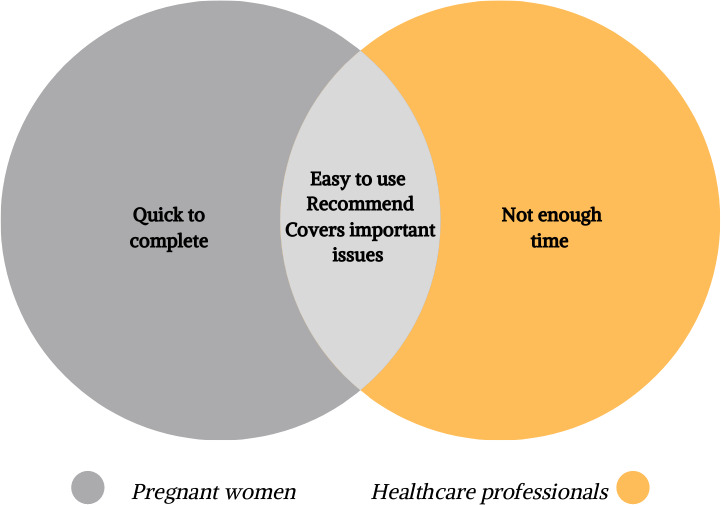
Attitudes of pregnant women and healthcare professionals to the FIGO Nutrition Checklist.

Figure 5 shows the similarities and differences in feedback on the use of the FIGO Nutrition Checklist in routine antenatal practice. All stakeholders agreed that it was easy to use and covered important issues for pregnancy. However, although the women felt that the Checklist was easy to complete, the healthcare professionals felt that there was insufficient time available to discuss it as part of the standard clinic appointment.

## 4 Discussion

The FIGO Nutrition Checklist is a brief nutritional questionnaire that is designed for use with all women before or during pregnancy. The purpose of the FIGO Nutrition Checklist is to collect simple information on weight, diet, and nutrition from women to support conversations on healthy lifestyle for pregnancy between women and their healthcare professionals and identify areas for improvement where relevant. Four in five women in this pilot reported that they did not meet at least one of the six dietary standards in the FIGO Nutrition Checklist, placing them at potential nutritional risk, despite the majority (92.4%) agreeing that they thought about their diet before the study. The findings of our study are in line with other research showing that there is poor adherence to pregnancy dietary recommendations, including intakes of vegetables, wholegrains, folic acid, and iron.^15^, ^16^ Fruit, vegetables, wholegrains, and fish are key components of the Dietary Approaches to Stop Hypertension (DASH) dietary pattern and following this diet has been associated with lower maternal blood pressure, even in women without hypertensive disorders.^17^

The most common dietary issue in our study was self‐reported fish intake of less than once a week. Fish, especially oily fish, is an important source of essential nutrients for pregnancy. These include iron, omega 3 fatty acids, and protein, a deficiency of which can negatively impact maternal health and fetal development.^18^ In this group, 34 (32.4%) women reported that they did not consume wholegrain carbohydrates at least once a day. Wholegrain carbohydrates are nutritionally superior to refined grains and tend to have a low glycemic index, a characteristic that has been associated with healthier gestational weight gain and glycemic control in pregnancy.^14^ The type and quality of carbohydrates consumed during pregnancy has also been associated with dietary micronutrient intakes.^19^ In addition, nearly one in five women (18.1%) reported that they did not consume fruits or vegetables at least 2–3 times a day. This standard is substantially lower than what is typically recommended for health, including during pregnancy.^20^ Low consumption of vegetables is associated with hypertensive disorders in pregnancy.^16^

Inadequate knowledge of dietary recommendations is one potential barrier for pregnant women to adhere to nutrition guidelines during pregnancy.^21^ While 83.3% (n=15) of the women who spoke with their healthcare professional about the FIGO Nutrition Checklist reported that they had the necessary information after this discussion, a lower percentage (66.7%, n=12) felt better prepared for behavior change after the consultation. In addition, while the majority (n=77, 73.4%) of women agreed that the FIGO Nutrition Checklist contains useful information, only 41 (39.0%) women reported learning something new from completing the Checklist. This highlights the importance of discussing the FIGO Nutrition Checklist as part of standard care, rather than providing it to women to use autonomously. It also suggests that motivation for behavior change, rather than lack of knowledge, is the barrier for some women. Research has shown that motivational interviewing techniques such as “healthy conversation skills” improve self‐efficacy for practitioners and can empower women for behavior change.^22^

The pregnant women in this pilot found the FIGO Nutrition Checklist was quick to complete autonomously; however, the obstetricians in this study felt that the FIGO Nutrition Checklist did not fit into their routine assessment owing to already limited clinic time. Despite this, two out of the three obstetricians agreed that it would be a beneficial tool for their practice and recommended it for use. We suggest that future work could, therefore, investigate how long completing and discussing the FIGO Nutrition Checklist takes in practice. The women in our study completed the Checklist before their appointment and strategies such as this may support maximum efficiency by protecting face‐to‐face clinical time for healthy conversations rather than questioning. Another option could be to explore the role of midwives and auxiliary staff in enhancing the efficiency of addressing nutrition in this way. The additional time investment needed for using the FIGO Nutrition Checklist should be considered by healthcare professionals in the context of the benefits to the women in their care, including capturing suboptimal dietary practices. Future work looking at the relationship between aspects of the FIGO Nutrition Checklist and relevant health outcomes, as done in a recently published study by Parisi et al.^23^, would be of interest to provide evidence to encourage healthcare professionals to implement the FIGO Nutrition Checklist in practice. As the FIGO Nutrition Checklist collects data on BMI and diet, it would be interesting for future studies to assess how its use in early pregnancy could affect or correlate with nutritional status and gestational weight gain throughout pregnancy.

This is the first study to assess the use of the FIGO Nutrition Checklist in practice. The strengths of the study include the multifaceted approach to investigate the acceptability of the FIGO Nutrition Checklist. A limitation is the small sample size of healthcare professionals. This was due to staff changeover and limited study clinics, which meant that very few doctors had enough exposure to the FIGO Nutrition Checklist to warrant feedback that would inform future implementation. The numbers surveyed, however, reflect the typical number of obstetricians that would be present at any one clinic in our maternity hospital. Furthermore, we did not survey any midwives in this study. Due to the nature of the study design and aims, we did not collect information on medical history or related data such as from blood tests. Future work looking at the relationship between FIGO Nutrition Checklist responses and relevant health outcomes would be of interest to provide evidence to encourage healthcare professionals to implement the FIGO Nutrition Checklist in practice.

We found that some women did not complete the questions on height, weight, and hemoglobin autonomously, suggesting that pregnant women attending outpatient clinics for routine care may not be aware of their own values or whether or not they have had them checked before. Therefore, completing the FIGO Nutrition Checklist may highlight to the woman that height, weight, and hemoglobin are important considerations for pregnancy and act as a reminder for the healthcare professional to measure and discuss these critical considerations with women at least once during pregnancy. We note that ACOG recommends the use of checklists in a clinical setting to support standardization of practice.^10^

Box 1 provides recommendations for adopting the FIGO Nutrition Checklist in different settings, based on our findings. The results of this study apply to the use of various dietary assessment tools and, in general, support this as part of routine practice. Previous research has found that incorporating new resources into clinical care requires a multitude of resources including an embedding approach, committed champions, and support from senior management members within the organization.^24^ However, an advantage of the FIGO Nutrition Checklist is the minimal resources for capacity building and training required, and hence it has high utility in resource‐poor settings.

Box 1 **Applying the FIGO Nutrition Checklist in a global context.**

- The FIGO Nutrition Checklist is a generic tool that can be adapted and localized based on the dietary and other needs of the population and practice setting.
- The providers of care in maternity services can vary across different countries or research settings. Involving midwives and other healthcare professionals in the use of the FIGO Nutrition Checklist can therefore be explored.
- We recommend researchers, clinicians, and public health practitioners to consider the local dietary habits and food‐based dietary guidelines. The content and questions of the checklist should be modified for each country (e.g. recommended calorie intake during pregnancy; questions related to meat consumption in countries with predominantly vegetarian diets).
- The guidelines for recording weight and body mass index of women during pregnancy may vary in countries, and hence gestational weight gain may not be discussed adequately. The FIGO Nutrition Checklist helps address this barrier.
- Resources for the low‐/middle‐income country context are also available in the FIGO Think Nutrition First report.^25^

Example: The UK has a midwifery‐based and community‐based model of antenatal care. In this case, the FIGO Nutrition Checklist would need to be adapted so that the content can be covered within the short duration of antenatal visits that already cover a wide range of issues. In addition, guidelines by the National Institute for Health and Care Excellence (NICE) differ from the information attached to the FIGO Nutrition Checklist for healthcare professional use on topics such as vitamin D/sun exposure and calorie intake during pregnancy. These can be modified to localize the FIGO Nutrition Checklist based on national guidelines.

In conclusion, the results of the present study show that the FIGO Nutrition Checklist is acceptable to pregnant women and that most women report some dietary practices that may put them at nutritional risk. Implementing the FIGO Nutrition Checklist as a screening tool is feasible for healthcare professionals but must be considered in the context of short antenatal consultations and visits. The FIGO Nutrition Checklist presents a brief, low‐cost intervention to guide healthcare professionals in the nutritional management of women during pregnancy so that they can promote their health and the health of future generations.

## Author Contributions

All authors were involved in the conception and design of the study. SLK and SC conducted the pilot study. SLK wrote the manuscript with input from all other authors. All authors provided input into the study design, analytical methods, and revisions of the manuscript.

## Conflicts of Interest

The authors have no conflicts of interest.

## Supporting information


**Supporting information S1**. FIGO nutrition checklist for pre‐pregnant/early pregnant women. Reproduced with permission from FIGO.
